# An Evaluation of Gastroscopy Findings During the COVID-19 Pandemic: A Public Hospital-Based Study From Turkey

**DOI:** 10.7759/cureus.46451

**Published:** 2023-10-04

**Authors:** Tuba Atak, Kıvılcım Orhun Erdoğan, Ibrahim Atak

**Affiliations:** 1 General Surgery, Bursa Çekirge State Hospital, Bursa, TUR; 2 General Surgery, Gaziosmanpaşa Research and Training Hospital, Istanbul, TUR; 3 General Surgery, Ali Osman Sonmez Oncology Hospital, Bursa, TUR

**Keywords:** helicobacter pylori, upper endoscopy, gastritis, pandemi, covid 19

## Abstract

Background and objective

Emotional factors can affect stomach secretions, acid expression, and stomach motor functions. The coronavirus disease 2019 (COVID-19) pandemic was an emotionally difficult time for many individuals as the whole world faced a life-threatening disease for which definitive treatment is still not fully established. In light of this, the aim of this study was to compare the results of gastroscopies performed among individuals before and after the pandemic.

Methods

The study included patients who underwent gastroscopy at Bursa Çekirge State Hospital in the following four different time frames: March-June 2019 (Group 1), March-June 2020 (Group 2), March-June 2021 (Group 3), and March-June 2022 (Group 4). All gastroscopy procedures were performed under anesthesia in the endoscopy unit. During the COVID-19 pandemic, all patients underwent a polymerase chain reaction (PCR) test, and gastroscopy was performed on those with negative results. Biopsies were taken from the antrum in all cases. Patient data were collected retrospectively and the groups were examined and compared in terms of age, gender, COVID-19 history, histopathology examination results, and diagnoses.

Results

A total of 803 patients were evaluated: 201 in Group 1, 200 in Group 2, 201 in Group 3, and 201 in Group 4. Group 1 comprised 66 (32.8%) males and 135 (67.2%) females, Group 2 consisted of 76 (38%) males and 124 (62%) females, Group 3 had 76 (37.8%) males and 125 (62.2%) females, and Group 4 comprised 86 (42.8%) males and 115 (57.2%) females. The mean age was 52.77 ±14.92 years in Group 1, 52.5 ±14.49 years in Group 2, 50.08 ±15.71 years in Group 3, and 52.83 ±13.20 years in Group 4. Helicobacter pylori (HP) positivity was found in 84 (41.8%) patients in Group 1, 146 (73%) in Group 2, 107 (53.2%) in Group 3, and 70 (34.8%) in Group 4. The HP infection was mild in 47 (23.4%) patients in Group 1, 26 (13%) in Group 2, 49 (24.4%) in Group 3, and 72 (35.8%) in Group 4. Moderate severity of HP infection was found in 16 (8%) patients in Group 1, 18 (9%) in Group 2, 25 (12.4%) in Group 3, and 25 (12.4%) in Group 4. Very severe HP infection was noted in 21 (10.4%) patients in Group 1, nine (4.5%) in Group 2, 20 (10%) in Group 3, and 34 (16.9%) in Group 4. With regard to mild HP infection, the highest rate was seen in Group 4 (35.8%). As for patients with very severe HP infection, a statistically significant difference was found between Group 2 and Group 4. In 2020 (Group 2), the rate was 4.5%, increasing to 16.9% in 2022 (Group 4). Regarding the comparison among groups based on histopathological examination findings, the frequency of chronic antral gastritis was determined to be highest in Group 4, at a statistically significant level (p<0.001).

Conclusion

The COVID-19 pandemic has caused physical and emotional hardships for several people worldwide. The possibility of transmission of the disease, unknown facts about the disease, and anxiety due to the condition being potentially fatal have had a drastic impact on the emotional states of many people. It is a condition that affects the lives of many people in the short term, and we believe that its effects reflected in the chronic period can be better evaluated through further studies conducted over the long term.

## Introduction

Emotional factors can affect stomach secretions, acid expression, and stomach motor functions among people [1-3]. The coronavirus disease 2019 (COVID-19) pandemic was an emotionally trying time for many individuals as the whole world reeled from a life-threatening disease for which definitive treatment methods are not fully established. In this study, we aimed to compare the results of gastroscopies performed among individuals before and after the pandemic.

## Materials and methods

Patients who underwent gastroscopy at the endoscopic unit of the general surgical clinic of Bursa Çekirge State Hospital were included in this study. The patients were classified into four groups as follows: Group 1 (pre-pandemic period: March-June 2019), Group 2 (first year of the pandemic: March-June 2020), Group 3 (second year of the pandemic: March-June 2021), and Group 4 (third year of the pandemic: March-June 2022). Patients who had previously received treatment for Helicobacter pylori (HP) infection were not included in the study. All gastroscopy procedures were performed under anesthesia in the endoscopy unit. During the COVID-19 pandemic, all patients underwent a polymerase chain reaction (PCR) test and gastroscopy was performed on those with negative results. Biopsies were taken from the antrum in all cases.

Patient data were collected retrospectively and the groups were examined and compared in terms of age, gender, COVID-19 history, histopathology examination results, and diagnoses. Ethical approval for the study was granted by the Bursa City Hospital Ethics Committee with the approval number 2022-13/6.

Statistical analysis

Data obtained in the study were analyzed statistically by using IBM SPSS statistics version 23.0 (IBM Corp., Armonk, NY). Conformity of the data to normal distribution was examined with the Shapiro-Wilk test. Descriptive statistics were presented as mean ±standard deviation (SD) for continuous variables and as number (n) and percentage (%) for categorical variables. In the comparisons of more than two groups of data with normal distribution, one-way analysis of variance (ANOVA) was used. For the analysis of categorical data, the Pearson Chi-square, Fisher-Freeman-Halton, and Fisher’s exact tests were used. When significance was determined, the Bonferroni test was used from the multiple comparison tests. The level of statistical significance was set at α=0.05.

## Results

A total of 803 patients were included in the final analysis: 201 in Group 1, 200 in Group 2, 201 in Group 3, and 201 in Group 4. Group 1 comprised 66 (32.8%) males and 135 (67.2%) females with a mean age of 52.77 ±14.92 years. Group 2 consisted of 76 (38%) males and 124 (62%) females with a mean age of 52.50 ±14.49 years. Group 3 had 76 (37.8%) males and 125 (62.2%) females with a mean age of 50.08 ±15.71 years. Group 4 comprised 86 (42.8%) males and 115 (57.2%) females with a mean age of 52.83 ±13.20 years. No statistically significant differences were observed between the groups in terms of age (p=0.182) and gender (p=0.237).

HP positivity was found in 84 (41.8%) patients in Group 1, 146 (73%) in Group 2, 107 (53.2%) in Group 3, and 70 (34.8%) in Group 4. Mild HP infection was seen in 47 (23.4%) patients in Group 1, 26 (13%) in Group 2, 49 (24.4%) in Group 3, and 72 (35.8%) in Group 4. Moderate severity of HP infection was determined in 16 (8%) patients in Group 1, 18 (9%) in Group 2, 25 (12.4%) in Group 3, and 25 (12.4%) in Group 4. Very severe HP infection was found in 21 (10.4%) patients in Group 1, nine (4.5%) in Group 2, 20 (10%) in Group 3, and 34 (16.9%) in Group 4 (Table 1).

**Table 1:** HP infection severity among the groups HP: Helicobacter pylori

Groups	HP positivity, %	Moderately severe HP infection, %	Very severe HP infection, %
Group 1: pre-pandemic period (March-June 2019)	41.8	8	10.4
Group 2: first year of the pandemic (March-June 2020)	73	9	4.5
Group 3: second year of the pandemic (March-June 2021)	53.2	12.4	10
Group 4: third year of the pandemic (March-June 2022)	34.8	12.4	16.9

With regard to patients documented to have mild HP infection, the highest rate was seen in Group 4 (35.8%). No significant difference was found between the groups with respect to moderate HP infection. As shown in Table 2, a statistically significant difference was seen between Group 2 and Group 4 in terms of very severe HP infection. In 2020 (Group 2), the rate was 4.5%, increasing to 16.9% in 2022 (Group 4). No statistically significant difference was observed between the groups with regard to the presence (p=0.095) and severity (p=0.311) of intestinal metaplasia or atrophy. Regarding the comparison among groups based on histopathological examination findings, the frequency of chronic antral gastritis was determined to be highest in Group 4, at a statistically significant level (p<0.001).

**Table 2:** Comparison of patient characteristics among groups +: mildly severe; ++: moderately severe; +++: very severe HP: Helicobacter pylori; IM: intestinal metaplasia CAG: chronic atrophic gastritis

Variable	Category	Group 1 (n=201)	Group 2 (n=200)	Group 3 (n=201)	Group 4 (n=201)	P-value
Age, years, mean ±SD		52.77 ±14,92	52.5 ±14.49	50.08 ±15.71	52.83 ±13.20	0.182
Gender	Male	66 (32.8%)	76 (38%)	76 (37.8%)	86 (42.8%)	0.237
	Female	135 (67.2%)	124 (62%)	125 (62.2%)	115 (57.2%)	
HP positivity	No	117 (58.2%)	147 (73.5%)	107 (53.2%)	70 (34.8%)	<0.001
	Yes	84 (41.8%)	53 (26.5%)	94 (46.8%)	131 (65.2%)	
Severity of HP	No	117 (58.2%)	147 (73.5%)	107 (53.2%)	70 (34.8%)	<0.001
	+	47 (23.4%)	26 (13%)	49 (24.4%)	72 (35.8%)	
	++	16 (8%)	18 (9%)	25 (12.4%)	25 (12.4%)	
	+++	21 (10.4%)	9 (4.5%)	20 (10%)	34 (16.9%)	
IM	No	182 (90.5%)	181 (90.5%)	184 (91.5%)	170 (84.6%)	0.095
	Yes	19 (9.5%)	19 (9.5%)	17 (8.5%)	31 (15.4%)	
Severitiy of IM	No	182 (90.5%)	181 (90.5%)	183 (91%)	170 (84.6%)	0.311
	+	15 (7.5%)	16 (8%)	15 (7.5%)	25 (12.4%)	
	++	3 (1.5%)	0 (0%)	2 (1%)	4 (2%)	
	+++	1 (0.5%)	3 (1.5%)	1 (0.5%)	2 (1%)	
Dysplasia	No	194 (96.5%)	194 (97%)	201 (100%)	201 (100%)	0.001
	Yes	7 (3.5%)	6 (3%)	0 (0%)	0 (0%)	
Atrophy	No	194 (96.5%)	194 (97%)	197 (98%)	200 (99.5%)	0,153
	Yes	7 (3.5%)	6 (3%)	4 (2%)	1 (0.5%)	
Diagnosis	Normal	14 (7%)	28 (14%)	23 (11,4%)	0 (0%)	<0.001
	CAG	187 (93%)	172 (86%)	178 (88.6%)	201 (100%)	
Severity of inflammation	No	14 (7%)	30 (15%)	23 (11,4%)	0 (0%)	<0.001
	+	58 (28.9%)	61 (30,5%)	61 (30.3%)	75 (37.3%)	
	++	69 (34.3%)	68 (34%)	51 (25.4%)	62 (30.8%)	
	+++	60 (29.9%)	41 (20.5%)	66 (32.8%)^a^	64 (31.8%)	

## Discussion

COVID-19 was first reported in Wuhan, China, on 29 December 2019, and it quickly spread from Asia to Europe and then the American continent within two months, causing the whole world to experience the most serious healthcare issue in recent history. The virus causing the pandemic was termed severe acute respiratory syndrome coronavirus 2 (SARS‑CoV‑2) [1]. WHO declared COVID-19 to be a global pandemic on 11 March 2020 [2,3]. Although timely responses and actions were implemented with regard to many problems related to the virus, many features still remain unclarified. In Turkey, the first case of COVID-19 was reported by the Ministry of Health on 10 March 2020 [4].

Emotional factors can affect stomach secretions and stomach motor functions among individuals; the expression of stomach acid increases, and a significant increase is seen in serum gastrin and pepsinogen levels [5]. HP infection, conditions increasing acid secretion, and the use of aspirin and non-steroidal anti-inflammatory drugs (NSAIDs) have been implicated in the formation of ulcers [3-5]. The first studies examining the relationship between emotional status and stomach acid were conducted by Beaumont et al. at the beginning of the 18th century [6]. In the current study, the highest rate of HP positivity in our cohort was recorded in the year when the pandemic started. This could be attributed to the increased stress that many individuals experienced at that time as little was known about COVID-19 back then; there were no vaccines, and many lives were being lost to the disease every day.

HP infection is the most common infection worldwide. It represents a serious concern as it is a major pathogen in gastritis, duodenal ulcers, stomach cancer, and mucosa-associated lymphoid tissue (MALT) lymphoma [3-6]. It is estimated that over half the world’s population is infected with HP, with a reported prevalence of 60% [7]. The rate of infection ranges from 5 to 15% in developed countries, whereas it varies between 36 and 82% in developing countries. The higher incidence in developing countries is linked to low socioeconomic conditions, poor healthcare, and crowded living conditions [7]. Vitamin and mineral deficiencies and poor nutrition lay the foundations for HP infection by disrupting stomach acidity [8]. In areas with overcrowded living conditions, as in many parts of Turkey, an increase in HP infection was observed during the COVID-19-related lockdown periods.

A study by Bozdağ et al. compared patients who underwent gastroscopy before and during the pandemic. The researchers found that rates of inflammation, intestinal metaplasia, and severe HP infection were significantly higher during the pandemic, whereas the rate of chronic gastritis was higher before the pandemic [9]. In the current study, the rate of chronic antral gastritis was significantly higher in the third year of the pandemic, which could be due to the fact that more people in our cohort were examined for this condition at a later period of the pandemic compared to the previous study.

HP positivity is a basic component in gastric atrophy, which is a precancerous condition. In the study by Bozdağ et al., the comparison between gastroscopies performed before and in the first year of the pandemic showed no significant difference between the groups with respect to atrophy [9]. Similarly, in the current study, no significant difference was found between the groups in terms of atrophy and intestinal metaplasia. We believe that for a more accurate evaluation of chronic conditions such as atrophy and intestinal metaplasia, the long-term effects of the pandemic should be examined.

As reported by international working groups, the long duration and persistence of the pandemic caused heightened concerns among people about unemployment and poverty [9-11]. In the current study, the difference in very severe HP infection was significantly high between Group 2 and Group 4. This could be ascribed to the increase in stress levels associated with COVID-19-related unemployment and economic problems and decreasing purchasing power.

Since the lungs and cells that make up the digestive apparatus and that secrete gastrin and other hormones produced in the stomach have the same embryological origin (endoderm), some studies have investigated if there is an association between HP infection and lung cancer [10]. A few studies have shown that patients infected with HP may be more prone to SARS-CoV-2 and experience a more severe form of COVID-19 [11-13]. There was no statistically significant difference between HP-positive and -negative patients in the current study in terms of their COVID-19 history. However, due to a lack of data, we could not examine aspects such as ICU admissions and disease conditions like viral pneumonia in HP-positive patients.

This study has a few limitations. Since the study was retrospective in design based on the data available in the medical records, factors such as the socioeconomic level of the patients and the number of people in patients' families could not be analyzed. Moreover, this study was conducted at a single center, which limits the generalizability of its findings to the broader population. Hence, we recommend further prospective studies and case series to gain deeper insights into the topic.

## Conclusions

COVID-19 continues to be a major health issue globally, as there are still several unknown factors surrounding it, and the pandemic has not fully ended. The unknown aspects related to the pathophysiology and treatment of the disease, combined with the financial implications and cost-of-living crises associated with the condition, have had a serious impact on people's emotional and mental well-being. We believe that the pandemic will increase the incidence of chronic antral gastritis and the severity of HP infections in the coming years. Hence, there is an urgent need for further long-term studies to investigate whether COVID-19 causes an increase in HP infections and whether the severity of COVID-19 is influenced by HP positivity.
